# Halitosis Relief Effect of Mouthwash Containing *Lespedeza cuneata* Extract: A Randomised, Double-Blind, Placebo-Controlled Study

**DOI:** 10.3290/j.ohpd.b4211059

**Published:** 2023-07-13

**Authors:** Yu-Rin Kim, Seoul-Hee Nam

**Affiliations:** a Professor, Department of Dental Hygiene, Silla University, Busan, Republic of Korea. Collected the data, drafted the initial manuscript, conducted the study.; b Professor, Department of Dental Hygiene, College of Health Sciences, Kangwon National University, Samcheok, Republic of Korea. Statistical analysis, critical revisions of the manuscript, supervised the study, study concept and design.

**Keywords:** bacteria, halitosis, *Lespedeza cuneata*, mouthwash, natural extracts

## Abstract

**Purpose:**

To evaluate the effect of a mouthwash containing *Lespedeza cuneata* extract (LCE) on halitosis as an alternative to chemical mouthwashes. The effect of this natural mouthwash on halitosis-causing bacteria (HCB) was clinically evaluated.

**Materials and Methods:**

A total of 84 subjects among 103 participants were recruited from the M Dental Clinic (Busan, South Korea) in this randomised, double-blind, placebo-controlled study. The participants were divided into two groups: 41 in the saline-gargle group (saline group) and 43 in the LCE-gargle group (LCE group). A week before the experiment, scaling and oral health education were conducted to standardise the subjects’ oral condition. For clinical evaluation, halitosis and HCB were evaluated pre-gargle (baseline), immediately after gargling (treatment), and 5 days after gargling (5 days post-treatment). Statistical analysis was performed using SPSS for Windows.

**Results:**

The level of subjective improvement was statistically significantly greater in the LCE group than the saline group (p < 0.05). Compared with the saline group, the LCE group showed reduced halitosis after 5 days of application. Furthermore, halitosis statistically significantly decreased over time (p < 0.05). Moreover, the LCE group showed a statistically significant decrease in the number of all six HCBs (p < 0.05).

**Conclusion:**

LCE gargle was effective in reducing halitosis both subjectively and objectively, suggesting an antibacterial effect on HCBs in the oral cavity. Thus, LCE, which can be used as a safe mouthwash ingredient, can promote oral health and will be valuable for the oral healthcare product industry. It might also be helpful for people suffering from halitosis.

Halitosis is defined as malodour exhaled through the mouth and nose, which can be unpleasant for the people around the affected individual.^31^ In fact, 85% of all cases of genuine halitosis which can be identified through the mouth, are caused by oral cavity issues, which are closely related to oral plaques, e.g. dental plaque, calculus, and debris on the tongue surface.^4^ Other causes include oral factors such as caries, periodontal diseases, soft tissue infection, oral cancer, oral candidiasis, poor dentures, prostheses, tongue disease, and dry mouth,^12^ as well as systemic diseases.^17,27^ As such, additional risk factors such as host genetics, lifestyle, stress, and systemic conditions dictate immune pathogenesis and are important for the transition from a healthy to a diseased status.^1^

If the equilibrium of the oral microflora is disturbed and maintained in an imbalanced state, an inflammatory reaction by microorganisms occurs, leading to oral diseases.^10^ Food debris, detached epithelial cells of the oral mucosa, and white blood cells from the periodontal pockets and saliva are decomposed by anaerobic gram-negative bacteria. As a result, volatile sulfur compounds (VSCs), such as methyl mercaptan and hydrogen sulfide, which cause halitosis, are formed by protein breakdown.^5^ *Porphyromonas gingivalis (P. gingivalis), Prevotella intermedia (P. intermedia), Prevotella nigrescens (P. nigrescens), Fusobacterium nucleatum (F. nucleatum), Veillonella alcalescens (V. alcalescens), Treponema denticola (T. denticola),* and *Campylobacter rectus (C. rectus)* cause halitosis.^3^ Therefore, effective antibacterial substances should be identified to reduce the bacterial activity. To prevent and ameliorate halitosis, the growth and proliferation of HCB in the oral cavity should be restricted.

Mouthwash is a typical chemical dental plaque control agent that can remove food residues remaining in the oral cavity and has been reported to have antibacterial effects against various bacteria that cause oral diseases.^19,32^ As an oral healthcare product, mouthwash has been used for several purposes, including oral disease care and halitosis improvement.^30^ Recently, the demand for mouthwash has increased, owing to halitosis resulting from mandatory mask wearing during the prolonged COVID-19 pandemic.^28^ Most mouthwashes sold in South Korea contain alcohol or chlorhexidine. Alcohol-containing mouthwash can cause xerostomia when used for a prolonged period.^26^ Furthermore, excessive use of these chemical mouthwashes causes side effects, such as bacterial infection, vomiting, and diarrhea, resulting from disturbed oral and intestinal microbiomes.^6^

Therefore, effective and safe antibacterial agents are actively being sought. Some natural antibacterial substances, such as phytoncides,^28^ seaweed extracts,^18^ and green tea extracts,^29^ can suppress HCB. Kim and Nam^16^ found that mouthwash containing *Sambucus williamsii* var. *coreana* extract effectively reduced halitosis as well as the number of HCB.

*Lespedeza cuneata* (LC), which belongs to the legume family, has been widely used as green manure; it also has various medicinal properties, e.g. antibacterial, antioxidant,^20^ anti-skin–photoaging,^13^ hypoglycemic,^14^ cell protection,^2^ insulin secretion stimulation,^2^ and wound healing.^11^ Despite this, no studies have been conducted on the clinical effects of LC on oral diseases. Therefore, this study aimed to evaluate the antibacterial effects of mouthwash containing LC as an alternative natural substance that inhibits harmful HCBs and ameliorates halitosis.

## Materials and Methods

### Extraction of LC

LC was purchased from Cheongmyeong (Goesan, Chungcheongbuk-do, South Korea). After adding 70% ethanol to crushed LC, it was extracted at 60°Ϲ for 12 h. LC extract (LCE) was filtered using qualitative filter paper and concentrated using a rotary vacuum evaporator (N-1300E.V.S. EYELA, Rikakikai; Tokyo, Japan). The extract was lyophilised using a freeze dryer at -80°C (Ilshin Lab; Yangju-si, South Korea). The sample was prepared as a powder and used as mouthwash containing 10 mg/ml LCE.

### Study Design and Protocol

This study was conducted as a randomised, double-blind, placebo-controlled trial. A dental hygienist with more than 10 years of experience directly explained the objective of the study to the patients who visited the M Dental Clinic in Busan from October 2020 to June 2021. Homogeneity of the oral conditions in all participants was ensured by light scaling after an oral examination by a dentist. The experiment was started 1 week after scaling to allow gum recovery, and instruction regarding toothbrushing and diet to avoid foods affecting halitosis (coffee, curry, onion, garlic, etc) was provided. The baseline was set at 1 week after scaling, and halitosis was measured before meals in the fasting state. To measure oral HCBs, the maxillary right first molars (#16) and mandibular left first molars (#36) were selected and analysed. Measurements were performed immediately after gargling, and the mouthwash provided was labeled so that it was not known whether the participant belonged to the experimental or control group. In the experimental group, the patients gargled with 15 ml of LCE for 30 s, whereas those in the control group gargled with 15 ml of saline for 30 s. Halitosis and HCB were measured after gargling. Thereafter, participants were instructed to use random gargles for 5 days before bed. Furthermore, oral health and dietary education was provided to ensure homogeneity of oral health behaviours among the participants as much as possible. After 5 days, halitosis and HCB were evaluated without oral hygiene before breakfast at the M Dental Clinic.

### Eligibility Criteria

#### Study participants

**Fig 1:**
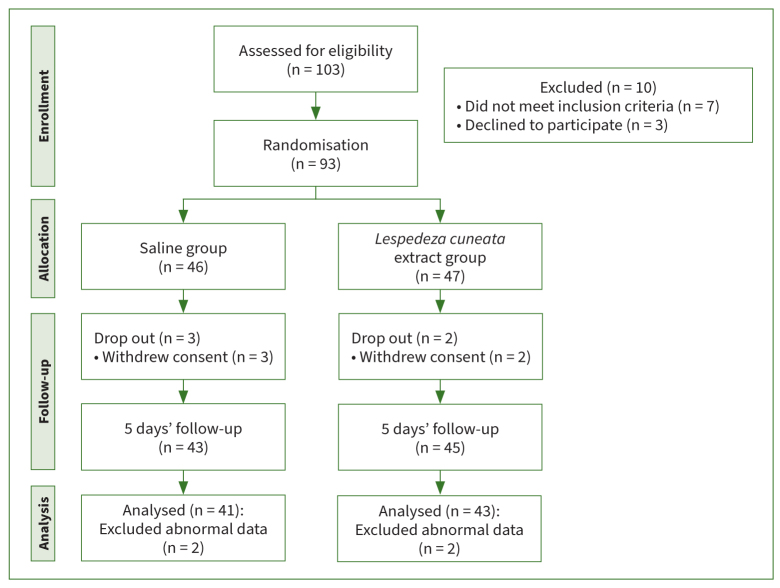
Flowchart of study method.

G* Power 3.1 (Heinrich Heine University; Düsseldorf, Germany) software was employed to calculate sample sizes. Sixty-eight (68) participants were required for an independent t-test with a significance level of α = 0.05 two-tailed, power = 0.8, and effect size = 0.7. The initially planned sample size was set at 96 to take a dropout rate of 40% into account, so that 103 individuals were chosen as potential participants. Of these, 10 patients with severe periodontitis, one or more dentinal carious lesions xerostomia, and currently receiving treatment for systemic diseases (liver disease, kidney disease, Sjögren’s syndrome, sinusitis, rhinitis) that can cause bad breath were excluded. Finally, 93 people were selected as study participants. The participants were randomly selected and divided into a saline group (46 participants) and an LCE group (47 participants). A final analysis was conducted on 84 participants, excluding those who withdrew from the study for personal reasons and those who showed abnormal results (Fig 1).

#### Inclusion criteria

Subjects who voluntarily signed an informed consent for this study were enrolled in the trial. Accordingly, the subjects of this study were those who agreed to complete the questionnaire, fulfilled no exclusion criteria, and had 16 or more remaining teeth with periodontal pocket depths of < 4 mm.

#### Exclusion criteria

Reasons for exclusion included severe dental disease (e.g. periodontitis, dry mouth, dental caries), systemic disease that may cause bad breath (e.g. Sjögren’s syndrome, rheumatism, renal disease, and hepatic disease), smoking, sinusitis or rhinitis, current antibiotic consumption, tongue problems (e.g., tongue cancer, glossitis), or having received scaling within two months prior to the study. Patients with enamel caries were eligible to participate in the study, but patients with more than one dentinal carious lesion were excluded. Thus, 10 patients were excluded, yielding 93 finally selected as study participants.

#### Withdrawals

Some participants discontinued the study due to personal reasons and others requested voluntary withdrawal.

### Clinical Measurement Tools

#### Questionnaire

The questionnaire included items related to age, marital status, systemic disease, and oral health behaviours, such as the frequency and timing of toothbrushing per day. Ten items – derived from modified and supplemented questionnaires from previous studies^6,8,13,22^ – were related to the subjective oral health status. Answers were given on a 5-point Likert scale, with a greater number of points indicating higher subjective oral health status. For halitosis, a 5-point scale was used to measure pseudo and subjective halitosis, with a higher score indicating stronger halitosis. The improvement in halitosis was investigated post-gargling in the two groups, with higher scores on the 5-point scale indicating greater improvement.

The reliability of the subjective oral-health status questionnaire was 0.319, and that of the halitosis questionnaire was 0.853.

#### Halitosis measurement

Two experienced dental hygienists measured halitosis three times, and the average value was recorded for accurate measurement. For a minimum of 5 min before measurement, VSCs were collected in the oral cavity by closing the mouth, and halitosis was measured without any oral hygiene performed in the morning, on an empty stomach. A Refres device (Mattz; Tokyo, Japan) was used to measure halitosis. Participants were asked to breathe only through the nose for 3 min, while halitosis was measured for 30 s by inserting a mouthpiece attached to the probe of the halitosis detector into the participant’s mouth. The readings were categorised as: no halitosis (up to 30 Bad Breath Value [BBV]); normal range (30–50 BBV); mild halitosis (up to 50 BBV); treatment required for halitosis (over 50 BBV); halitosis present (up to 70 BBV); distinct halitosis (up to 90 BBV); and severe halitosis (100 BBV).

### Microbiological Analysis

The maxillary right first molar (#16) and mandibular left first molar (#36) were selected to measure HCB. A paper point no. 15 was inserted into the periodontal pocket for 10 s to collect bacteria. After collecting, bacteria were placed in 1.5-ml sterilised tubes and stored at -20°C before the analysis. DNA from the collected paper points was extracted using the AccuPrep Universal RNA Extraction Kit (Bioneer; Daejeon, Korea). Extraction was performed according to the manufacturer’s instructions. OligoMix (YD Global Life Science; Seongnam, Korea) and three oligonucleotides (forward primer, reverse primer, and probe; Table 1) that react specifically to each bacterium were used.^19^ To prepare the polymerase chain reaction (PCR) sample, 9 μl of OligoMix, 10 μl of 2x probe qPCR mix (Takara Bio; Shiga, Japan), and 1 μl of template DNA were combined. A 96-well plate containing the PCR reaction sample was placed in the CFX96 Touch Real-time PCR Detection System (Bio-Rad; Hercules, CA, USA) for DNA amplification. The PCR conditions were as follows: initial denaturation at 95°C for 30 s, denaturation at 95°C for 10 s, and annealing for 30 s at 62°C with 40 repeated cycles. The cycle threshold (Ct) value was calculated using Bio-Rad CFX Manager software, and the number of copies was derived by plotting the Ct value in the standard curve of each bacterium (Table 1).

**Table 1:** Primers and probes used in the real-time PCR assays

Bacterium	Target genes	Primers/Probe sets	Amplicon size (bp)
*Porphyromonas gingivalis*	hemagglutinin (phg) gene	5′-ACACGGTGTATCGTGACGGC-3’ 5′-GCCGGCTGCGTACTTAACCT-3’ 5′-HEX-CGACCTACCGCGATGCAGGA-BHQ1–3’	119
*Treponema denticola*	oligopeptidase B (opdB) gene	5′-AGAAAGGCTTTGGGCGACAG-3’ 5′-GCTGGAGCCGTAGCTTCCAT-3’ 5′-Cy5-CGGGTCCTCACCCGCTCTTC-BHQ2–3’	127
*Fusobacterium nucleatum*	16S ribosomal RNA gene	5′-GGCTGTCGTCAGCTCGTGTC-3’ 5′-CTCATCGCAGGCAGTATCGC-3’ 5′-FAM-AACGAGCGCAACCCCTTTCG-BHQ1–3’	114
*Prevotella intermedia*	hemagglutinin (phg) gene	5′-CACACGCTGGCGAAACCTAC-3’ 5′-CACGTGGCGTTGCTTCTTTC-3’ 5′-HEX-CCGAAGATGCGCCGTTGAAC-BHQ1–3’	143
*Prevotella nigrescens*	gyrase subunit B (gyrB) gene	5′-AGCAAGCTGTAGGCGAGGCT-3’ 5′-GCTGAACACTTTCGCGTGCT-3’ 5′-Texas Red-GCTCGTATTGCAGCCCGCAA-BHQ2–3’	132
*Campylobacter rectus*	groEL gene	5′-AAATTTAAGCGGCGACGAGG-3’ 5′-TCCTTGCTCACGCTTACGGA-3’ 5′-HEX-GGCTTTGACGCGGGCGTAGT-BHQ1–3’	132

### Ethics Approval and Consent to Participate

This study was conducted in accordance with the International Council for Harmonisation of Technical Requirements for Pharmaceuticals for Human Use (ICH) guidelines, reviewed by the Research Ethics Review Committee of Kangwon National University (KWNUIRB-2020-07-007-002, Chuncheon, South Korea), and registered as a clinical trial in the WHO International Clinical Trial Registry Platform (ICTRP) (registration date: 13/06/2022; registration number: KCT0007379; https://cris.nih.go.kr/cris/search/detailSearch.do/22017). Informed consent was obtained from all participants prior to their participation in the study. Participants were informed that they could withdraw from the study at any time without penalty. Furthermore, all identifying data were destroyed or not collected, and sufficient explanation of the ethical aspects was provided to all participants.

### Statistical Analysis

All data were analysed using SPSS 21.0 for Windows (IBM; Armonk, NY, USA). Normality was confirmed, with Skewness 0.613±0.263 and Kurtosis -1.187±0.520. Independent t-tests and Fisher’s exact tests were conducted to determine the differences in demographic characteristics, oral hygiene behaviours, and oral health status between the saline and LCE groups. An independent t-test and one-way ANOVA were performed to evaluate changes in halitosis and HCB between the two groups at baseline, at the treatment timepoint, and 5 days post-treatment. In addition, Tukey’s test was performed as a post-hoc test for these three timepoints, with the significance level set at p = 0.05 as a two-tailed test.

## Results

### Study Population

In both the saline and LCE groups, there were more females than males; the average age was 29.2 years in the saline group and 26.7 years in the LCE group. Regarding marital status, there were more singles in both groups. There were more participants without systemic diseases in both groups. None of the variables showed significant differences between the two groups (Table 2).

**Table 2:** Characteristics of the subject in the saline and LCE groups

Characteristics			N (%)		p-value
			Saline group (n = 41)	LCE group (n = 43)	
*Gender	Male		9 (22.0)	6 (14.0)	0.339
	Female		32 (78.0)	37 (86.0)	
^￥^Age (mean ± SD)			29.22 ± 8.49	26.67 ± 7.77	0.155
*Marriage		Single	32 (78.0)	37 (86.0)	0.339
		Married	9 (22.0)	6 (14.0)	
*Systemic disease		No	36 (87.8)	40 (93.0)	0.478
		Yes	5 (12.2)	3 (7.0)	

^￥^Independent t- test; *Χ^2^ test, (p < 0.05). Values are means ± standard deviations.

### Group Differences in Oral Health Behaviour, Oral Health Status, and Halitosis

Regarding oral health behaviour, the frequency of toothbrushing was higher in the saline than in the LCE group. Furthermore, participants in the LCE group brushed their teeth more frequently after breakfast and before bed, whereas those in the saline group brushed more frequently after dinner. The frequency and timing of brushing did not differ statistically significantly between the groups. In terms of oral health status, no statistically significant differences were noted in any variable between the groups. Furthermore, the presence and severity of halitosis were not statistically significantly different between the groups. On the other hand, compared to the saline group, the improvement in halitosis was statistically significantly higher in the LCE group (p < 0.05; Table 3).

**Table 3:** Oral health behaviour and oral health status according to the presence or absence of pseudo-halitosis

	Questionnaire items	Saline group (n = 41)	LCE group (n = 43)	*p-value
Oral health behaviour	Timepoints of daily toothbrushing	3.71 ± 0.46	3.67 ± 0.47	0.748
	^￥^I brush my teeth before breakfast (n = 58)	29 (50.0)	29 (50.0)	0.744
	^￥^I brush my teeth after breakfast (n = 35)	15 (42.9)	20 (57.1)	0.356
	^￥^I brush my teeth after lunch (n = 58)	29 (50.0)	29 (50.0)	0.744
	^￥^I brush my teeth after dinner (n = 25)	13 (52.0)	12 (48.0)	0.703
	^￥^I brush my teeth before going to bed (n = 68)	31 (45.6)	37 (54.4)	0.223
Oral health status	I have good oral health	3.68 ± 0.96	3.58 ± 0.88	0.614
	I’ve never had a hard time chewing food	4.63 ± 0.62	4.63 ± 0.69	0.965
	I have never had swollen or bleeding gums	3.29 ± 1.21	3.42 ± 1.22	0.636
	I don’t bleed even when I brush my teeth	3.54 ± 0.93	3.63 ± 0.98	0.661
	I like to eat cold or hot food	4.49 ± 1.08	4.40 ± 1.30	0.723
	I don’t have dry mouth	4.15 ± 1.09	3.91 ± 1.27	0.357
	I have no halitosis	3.56 ± 0.84	3.42 ± 0.76	0.418
	I have correct pronunciation	3.73 ± 1.40	3.60 ± 1.45	0.684
	I don’t have any discomfort in the jaw joint	4.27 ± 0.74	4.12 ± 0.82	0.377
	I am not concerned about my oral health	4.00 ± 0.92	3.79 ± 0.99	0.319
Halitosis	^￥^I have halitosis (n = 69)	34 (49.3)	35 (50.7)	0.969
	Severe degree of halitosis	1.29 ± 0.81	1.30 ± 0.77	0.956
	^￥^I have improved halitosis (n = 51)	15 (29.4)	36 (70.6)	**0.000**
	Degree of improvement in halitosis	1.83 ± 0.86	4.14 ± 0.52	**0.000**

*Independent t-test; ^￥^Fisher’s exact test (p < 0.05). Values are means ± standard deviations. Boldface: statistically significant.

### Halitosis Difference in the Two Groups over Time

Regarding the halitosis levels in the saline and LCE groups, there were no statistically significant differences at baseline; however, a statistically significant difference was observed in the treatment group after 5 days (p < 0.05). Furthermore, during the 5 days of application, the saline group did not show any statistically significant difference, only a slight decrease (p>0.05), whereas the LCE group showed a statistically significant decrease (p>0.05; Table 4).

**Table 4:** Changes in halitosis according to the application of gargle

Variables	Baseline	Treatment	5 days post-treatment	*p-value
Saline group	44.00 ± 11.80^a^	42.04 ± 10.74^a^	38.46 ± 8.19^a^	0.639
LCE group	45.80 ± 15.93^a^	19.33 ± 5.21^b^	16.44 ± 6.07^b^	**0.000**
^￥^p-value	0.836	**0.000**	**0.000**	

^￥^Independent t-test; * one-way ANOVA and Tukey’s test (p < 0.05). Values are means ± standard deviations. Boldface: statistically significant. Different superscript letters indicate the statistically significant parameters.

### Change in HCB over Time

The numbers of *P. gingivalis, T. denticola, P. intermedia, P. nigrescens,* and *C. rectus* showed a significant difference between treatment and 5 days post-treatment in the LCE group. Furthermore, a statistically significant difference was noted in the number of *F. nucleatum* after 5 days (p < 0.05). Moreover, during the 5 days, the saline group showed a statistically significant decrease in the number of *P. gingivalis* and *P. nigrescens* over time, whereas the LCE group showed a statistically significant decrease for all bacteria (Table 5).

**Table 5:** Difference in halitosis-causing oral bacteria according to the application of gargle

Variables		Baseline	Treatment	5 days post-treatment	*p-value
*Porphyromonas gingivalis*	Saline group	1841.83 ± 1332.01^a^	2028.76 ± 554.61^a^	870.09 ± 326.18^b^	**0.009**
	LCE group	2525.20 ± 2148.79^a^	131.70 ± 213.49^b^	45.78 ± 76.17^b^	**0.000**
	^￥^p-value	0.344	**0.000**	**0.000**	
*Treponema denticola*	Saline group	10033.35 ± 18828.26^a^	844.65 ± 713.33^a^	593.06 ± 421.60^a^	0.057
	LCE group	6588.55 ± 9739.80^a^	322.78 ± 458.80^b^	114.78 ± 135.71^b^	**0.002**
	^￥^p-value	0.562	**0.032**	**0.000**	
*Fusobacterium nucleatum*	Saline group	875248.89 ± 345484.65^a^	633218.55 ± 394407.33^a^	650226.94 ± 365291.47^a^	0.194
	LCE group	1549395.06 ± 1022621.42^a^	554689.79 ± 441549.02^b^	314468.92 ± 361364.54^b^	**0.000**
	^￥^p-value	0.058	0.378	**0.005**	
*Prevotella intermedia*	Saline group	1648.07 ± 1126.31^a^	1449.91 ± 622.24^a^	1057.89 ± 464.34^a^	0.253
	LCE group	3283.53 ± 2870.58^a^	13.58 ± 21.42^b^	12.77 ± 18.80^b^	**0.000**
	^￥^p-value	0.080	**0.000**	**0.000**	
*Prevotella nigrescens*	Saline group	27535.58 ± 25528.61^a^	13426.62 ± 10839.14^a,b^	8283.22 ± 7049.11^b^	**0.031**
	LCE group	25092.80 ± 27018.24^a^	2364.16 ± 2310.57^b^	1253.47 ± 1180.49^b^	**0.000**
	^￥^p-value	0.841	**0.002**	**0.003**	
*Campylobacter rectus*	Saline group	25529.36 ± 25041.59^a^	16583.64 ± 22111.24^a^	7883.00 ± 8570.47^a^	0.121
	LCE group	29721.80 ± 27758.61^a^	228.55 ± 367.09^b^	5.71 ± 9.63^b^	**0.000**
	^￥^p-value	0.676	**0.015**	**0.006**	

^￥^Independent t-test; *one-way ANOVA and Tukey’s test (p < 0.05). Values are means ± standard deviations. Boldface: statistically significant. Different superscript letters indicate the statistically significant parameters.

## Discussion

Halitosis is an oral health problem that can act as a major obstacle in the affected individual’s social life. As the overall quality of life improves, interest in the treatment of halitosis is also increasing. The use of mouthwash has increased as a simple and readily available method that can be applied when it is difficult to brush teeth mechanically.^23^ Therefore, in this study, LCE was explored as an alternative natural ingredient in a mouthwash solution. The medicinal effect of LC includes protecting liver and kidney function, strengthening lung function and blood circulation. Additionally, it has been reported to have an anti-photoaging and skin-lightening effect.^2,11,13,14,20^ In this study, 10 mg/ml LCE was used, which was confirmed to have an antibacterial effect against HCB without cytotoxicity,^24^ suggesting the possibility of its safe use as a natural antibiotic. Therefore, we attempted to confirm the antibacterial and anti-halitosis effects of a safe LCE-containing mouthwash for clinical dental application.

Although there was no statistically significant difference in the baseline halitosis levels between the two groups, halitosis levels decreased with treatment and after 5 days in the LCE group. Furthermore, compared with the saline group, which did not show any statistically significant difference, the LCE group showed a significant decrease in halitosis levels when measured over 5 days. According to previous study,^8^ mouthwash containing *Phellodendron amurense* bark and *Machilus thunbergia* statistically significantly improved halitosis at 60 min, and *Paeonia suffruticosa Andrews* root bark significantly improved halitosis at 40 min after gargling. However, these results were analysed for only up to 1 h; therefore, the results of the present study are highly relevant, as it investigated the effect for 5 days, confirming the safety of prolonged use of the tested mouthwash.

Furthermore, Yoon et al^33^ reported that the subjective perception of halitosis decreased after 1 week of use of mouthwash containing cinnamon. Similarly, in the present study, the subjective perception of halitosis improvement in the saline group was scored as 1.83±0.86 points, whereas that in the LCE group received a relatively high score at 4.14±0.52, proving the anti-halitosis effect, and indicating statistically significantly improved halitosis levels perceived directly by the participants.

Furthermore, the mouthwash containing LCE showed a clear effect against HCBs *P. gingivalis, T. denticola, P. intermedia, P. nigrescens, C. rectus,* and *F. nucleatum*. Although the number of *F. nucleatum* indicated a statistically significant difference after 5 days of application, that of other bacteria decreased statistically significantly immediately after gargling, resulting in a decrease in the numbers of all bacteria after 5 days of LCE gargle application, which confirms the antibacterial effect of LCE. The typical bacteria that cause halitosis associated with periodontal disease, *P. gingivalis* and *P. intermedia*, produce methyl mercaptan and hydrogen sulfide from serum proteins.^6^ Compared with the baseline numbers, *P. gingivalis* and *P. intermedia* decreased by 98.2% and 99.6%, respectively, after 5 days of LCE gargle use, proving the natural antibacterial clinical effect of LCE.

Among commercially available mouthwashes, chlorhexidine and cetylpyridinium chloride are representative of those with chemical components, which may cause bacterial drug resistance when used longer time.^23^ To avoid such problems, several natural ingredients have been the subject of research. However, actual mouthwashes with natural ingredients are seldom developed, as most natural extracts have disadvantages such as the lack of efficacy when compared with synthetic chemical mouthwashes, price, usability with existing ingredients, and toxicity. However, LC is widely distributed as well as easy to obtain and prepare, making it a valuable ingredient that can be used in oral industrial products. The limitation of this study is that it was conducted over a short period of 5 days, so it is difficult to generalise the results. Therefore, studies with long-term follow-ups are needed. In addition, when measuring bad breath, foods that cause bad breath (garlic, onion, etc.) were restricted; however, since the study subjects lived at home, they would not have been able to completely control their diet for 5 days. Nevertheless, an LCE mouthwash is safe and plant-based, able to reduce the number of oral HCB. Its efficacy against halitosis has been clinically demonstrated here. Therefore, LCE mouthwash is expected to have high commercial applicability as a natural anti-halitosis oral-care product.

## Conclusion

As no previous studies have reported the antibacterial and anti-halitosis effects of LCE, this study’s findings can be used as industrially valuable data for developing natural antibacterial mouthwashes. Thus, as an alternative mouthwash, LCE-containing mouthwash can contribute to an improved oral milieu and thus oral health, resolving the problems related to the side effects of chemical mouthwashes.
